# Atorvastatin increases the production of proinflammatory cytokines and decreases the survival of *Escherichia coli*-infected mice

**DOI:** 10.1038/s41598-019-48282-2

**Published:** 2019-08-12

**Authors:** Hadi M. Hussein, Diva Kalash Al-Khoury, Alexander M. Abdelnoor, Elias A. Rahal

**Affiliations:** 1 Department of Experimental Pathology, Immunology and Microbiology, American University of Beirut, Beirut, Lebanon; 2 Center for Infectious Diseases Research (CIDR), American University of Beirut, Beirut, Lebanon

**Keywords:** Infection, Bacteriology, Chemokines, Infectious diseases

## Abstract

To assess whether the immunosuppressive effects of atorvastatin outweigh its antibacterial ones in an infection, mice were infected with *Escherichia coli* and administered atorvastatin; survival rates were then monitored. Mice treated with atorvastatin post-infection showed a remarkable decrease in their survival rate. On the other hand, the higher the level of serum IFN-γ in the infected mice treated with atorvastatin, the lower was the survival rate. Levels of IL-4 were markedly depressed in all groups infected with *E. coli* and treated with atorvastatin. Since atorvastatin inhibits IFN-γ expression in the absence of bacterial infection, we examined whether bacterial lipopolysaccharide (LPS) was the element capable of overriding this inhibition. Mouse peripheral blood mononuclear cells were treated with atorvastatin and lipopolysaccharide *ex vivo* then proinflammatory (IFN-γ, TNFα, IL-6) and prohumoral/regulatory (IL-4, IL-13, IL-10) cytokine levels were analyzed in culture supernatants. While proinflammatory cytokine levels were decreased upon treatment with atorvastatin alone, their levels were markedly elevated by treatment with LPS, bacterial lysate or bacterial culture supernatant. On the other hand, atorvastatin exerted an inhibitory effect on production of the prohumoral/regulatory cytokines. Our data indicates that any consideration for statins as antimicrobial treatment should assess the possible adverse outcomes.

## Introduction

A myriad of immunomodulatory and anti-inflammatory effects have been attributed to the statins class of antidyslipidemia agents. These 3-hydroxy-3-methylglutaryl coenzyme A (HMG-CoA) reductase inhibitors are widely used in the prevention of cardiovascular disease; however, numerous non-lipid lowering related effects have been reported for these agents. As a result of competitively inhibiting HMG-CoA reductase, the rate-limiting enzyme of the mevalonate pathway, statins decrease the production of farnesyl pyrophosphate and geranylgeranyl pyrophosphate which in turn prevents appropriate cell membrane association and signaling of various small GTPases^1^; this ultimately results in affecting various immune cell functions such as antigen presentation^2^, proinflammatory cytokine expression^3^, phagocyte function^4^ and T cell capabilities^5^. Additionally, statins reduce the production of mevalonate which has been reported to play a key role in immune training^6^, the rather recently described capability of the innate arm of the immune system to build an immune memory^7^.

Numerous studies have detected antimicrobial effects for statins both *in vitro* and in animal models. The direct antifungal effects of statins are reliant on the inhibition of the fungal HMG-CoA reductase but seem to vary between species regarding their mevalonate pathway metabolite dependency; effects for the decrease in ergosterol levels^8,9^, protein isoprenylation inhibition^10^ and iron uptake impairment^11^ have been indicated. On the other hand, mechanisms underlying the direct antibacterial effects of statins remain unclear. The affinity of bacterial HMG-CoA reductase to statins is 10^4^-fold less than the eukaryotic counterpart^12^. Moreover, statins have been reported to impair the growth of bacterial species that possess HMG-CoA reductase and those that do not to a similar extent. Despite the reported antimicrobial effects of statins, clinical data from patients have shown conflicting outcomes. While some studies and meta-analysis reviews have shown improved infection outcomes, others have shown otherwise; this may be attributed to possible sample size and study biases (reviewed in^13^).

To examine this dynamic and assess whether the immunosuppressive effects of statins are more relevant than its antimicrobial effects, we have previously examined an atorvastatin-treated *Candida albicans* mouse model of infection^14^. Treatment of mice with this statin before or after infection resulted in a reduced survival rate and in decreased expression of markers of proinflammatory and prohumoral immune responses. In light of the debatable reported improved clinical outcomes of statin treatment in bacterial infections, we herein examine whether the effects of statins in such an infection differ from those of a fungal one in a mouse model and determine whether particular immune components are affected differently.

## Results

### Administering atorvastatin to mice post-infection with *Escherichia coli* reduces their survival rate

Atorvastatin has been documented to have immunosuppressive effects; however, it also seems to have antibacterial effects that have been well demonstrated using *in vitro* assays. We hence examined whether the immunosuppressive effects of this statin would overcome its antibacterial properties in the setting of a systemic bacterial infection. BALB/c mice were infected with *E. coli* and administered atorvastatin either repeatedly for 4 days prior to infection, a single dose on the day of infection or repeatedly for 4 days after infection. Mice that were given repeated doses of the statin also received a dose on the day of infection.

**Figure 1:**
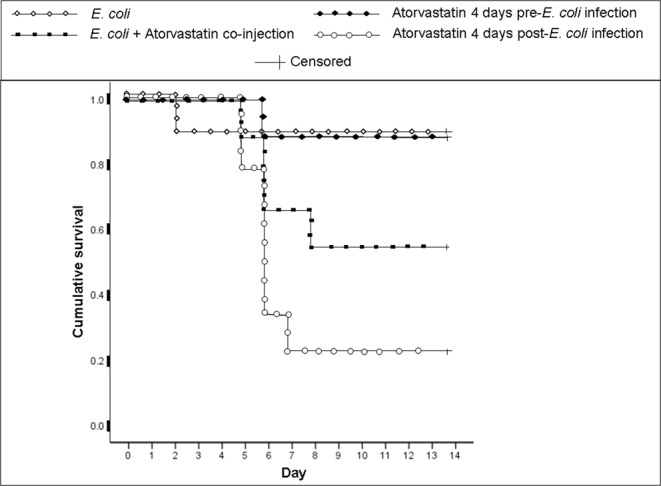
Effect of atorvastatin treatment on the survival of *E. coli*-infected mice. BALB/c mice (9 per group) were intraperitoneally injected with *E. coli* and treated either with a single co-injection of atorvastatin, a 4-day pre-infection or a 4-day post-infection atorvastatin regimen. A group infected with *E. coli* but not treated with atorvastatin was also included. Day 0 indicates the day of infection. Mice were monitored for 14 days after infection. The remaining number of mice at the end of the monitoring period was 2 in the group treated with atorvastatin for 4 days post-infection, 4 in the group administered a single dose of atorvastatin upon infection, and 8 in both the group treated with atorvastatin for 4 days pre-infection or infected but not treated with atorvastatin.

Survival analysis (p = 0.01) showed that the lowest survival rate, 22.2%, was observed in the group treated with atorvastatin for 4 days post-infection (Fig. 1). In this group, 2 mice died on day 4 post-infection, 4 mice died on day 5 post-infection, and 1 mouse died on day 6 post-infection; hence, 2 mice remained by the end of the monitoring period. The group administered a single dose of atorvastatin upon infection had a survival rate of 55.6%. In this group, 1 mouse died on day 4 post-infection, 2 mice died on day 5 post-infection and 1 mouse died on day 7 post-infection; hence, 4 mice remained by the end of the monitoring period. The survival rate of the group treated with atorvastatin for 4 days pre-infection was 88.9% whereby 1 mouse died on day 6 post-infection; hence, 8 mice remained by the end of the monitoring period. The survival rate of the group infected but not treated with atorvastatin at the end of the monitoring period also was 88.9% whereby 1 mouse died on day 2 post-infection; hence, also 8 mice remained by the end of the monitoring period.

Spleens, hearts and livers were collected from mice upon death and examined for infection and injury. Organs from infected atorvastatin-treated and non-treated animals showed a similar burden of infection and extent of injury. Hence, while the incidence of infection was enhanced by atorvastatin treatment, the severity of infection was not affected.

### Administration of atorvastatin markedly increases serum IFN-γ levels in *E. coli*- infected mice but decreases those of IL-4

**Figure 2:**
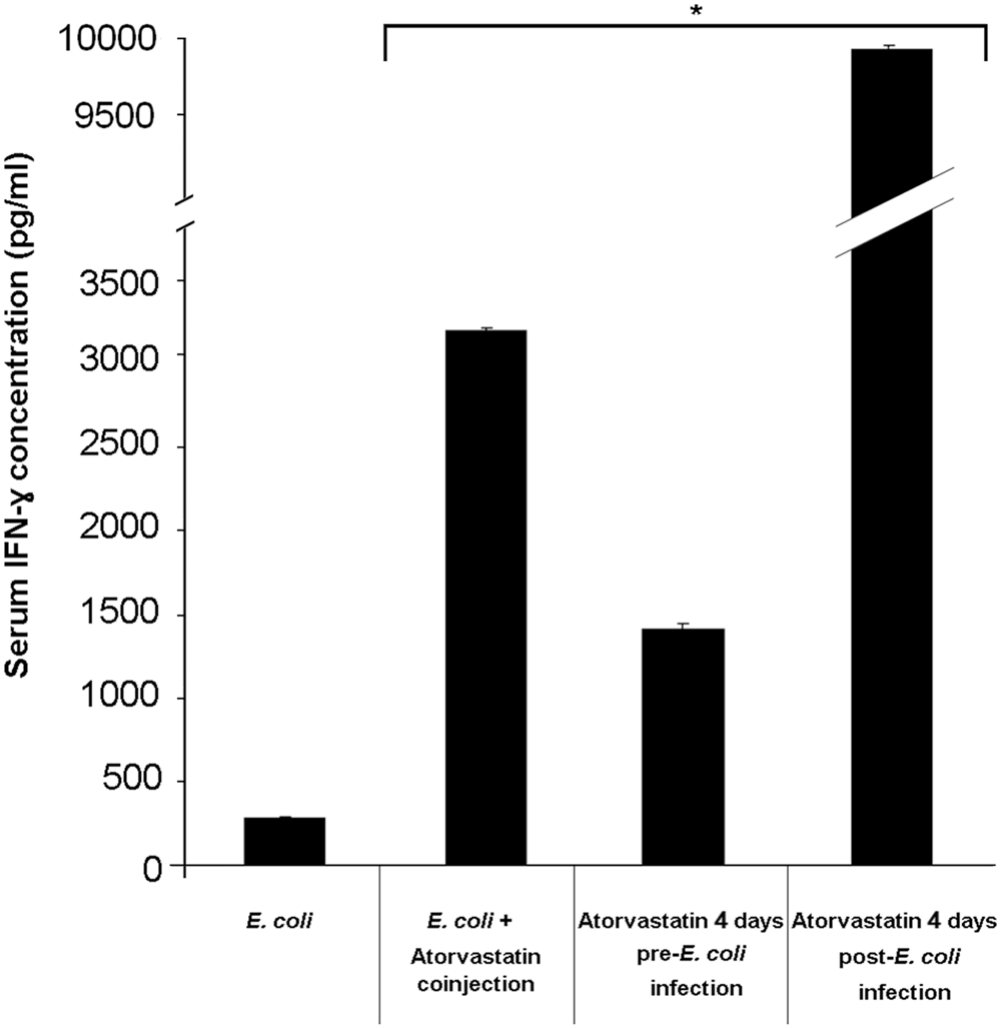
Serum IFN-γ levels in mice infected with *E. coli* and treated with atorvastatin. BALB/c mice (9 per group) received an intraperitoneal injection *E. coli* and were treated either with a single co-injection of atorvastatin, a 4-day pre-infection or a 4-day post-infection atorvastatin regimen. A group that was infected with *E. coli* but not treated with atorvastatin was included as well. Mouse sera were analyzed on day 4 after infection for IFN-γ levels by ELISA. *indicates p < 0.05 compared to the *E. coli*-infected group.

With the largest number of mouse death events observed on day 5 post-infection, we examined mouse cytokine levels one day prior. Mice infected with *E. coli* and treated with atorvastatin all displayed a significant increase in the levels of IFN-γ, the proinflammatory cytokine, on day 4 post-infection compared to the group infected with *E. coli* but not treated with atorvastatin (Fig. 2). The highest increase was observed in the group treated with atorvastatin for 4 days post-infection; a 35-fold increase was noted in this group (p < 0.0001). This group concomitantly had the lowest survival rate among the *E. coli*-treated groups. The second highest increase was in the group treated with a single dose of atorvastatin on the same day as receiving the infection; an 11-fold increase was detected in this group (p < 0.0001). This group also had the second lowest mouse survival rate. The group with the lowest serum IFN-γ increase, a 5-fold one (p < 0.0001), was that pre-treated with atorvastatin for 4 days prior to infection; this group also had the highest survival rate. Therefore, the survival rates of the groups that were infected with *E. coli* and treated with atorvastatin were largely mirrored by their serum IFN-γ levels whereby the higher the levels of this cytokine the lower was the noted survival rate.

**Figure 3:**
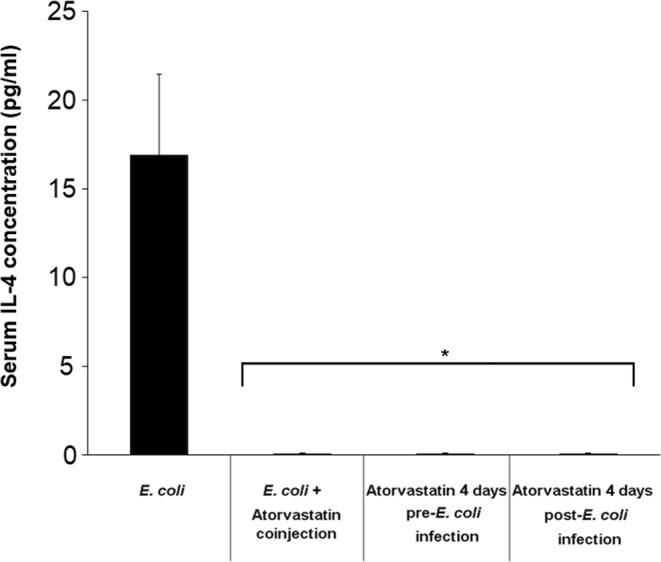
Serum IL-4 levels in mice infected with *E. coli* and treated with atorvastatin. BALB/c mice (9 per group) received an intraperitoneal injection *E. coli* and were treated either with a single co-injection of atorvastatin, a 4-day pre-infection or a 4-day post-infection atorvastatin regimen. A group that was infected with *E. coli* but not treated with atorvastatin was included as well. Mouse sera were analyzed on day 4 after infection for IL-4 levels by ELISA. *indicates p < 0.05 compared to the *E. coli*-infected group.

All mouse groups infected with *E. coli* and treated with atorvastatin showed a significant suppression of serum IL-4 levels compared to the group infected but not treated with the statin (Fig. 3). A 17-fold decrease in the level of this prohumoral immune response cytokine was detected in the atorvastatin-treated groups (p = 0.0043).

### Treatment of mouse pheripheral blood mononuclear cells with atorvastatin upon or after incubation with bacterial lipopolysaccharide significantly enhances their release of proinflammatory cytokines

We had previously observed that atorvastatin decreases the expression of IFN-γ and IL-4 levels in egg albumin-challenged mice^15^ and reduces serum IFN- γ and IL-4 in *C. albicans*-infected mice^14^. With lipopolysaccharide  (LPS) being a highly proinflammatory bacterial component, we examined whether it could overcome the atorvastatin-mediated inhibition of IFN-γ production as we had observed in *E. coli*-infected mice in addition to examining other mediators of the proinflammotory and prohumoral/regulatory responses. For this purpose, mouse pheripheral blood mononuclear cells (PBMCs) were treated with LPS alone and received a co-treatment, pre-treatment or post-treatment with atorvastatin. Alternatively, PBMCs were incubated with an amount of *E. coli* cell lysate or *E. coli* culture supernatant containing an equivalent quantity of LPS to that used alone in addition to the atorvastatin treatment.

**Figure 4:**
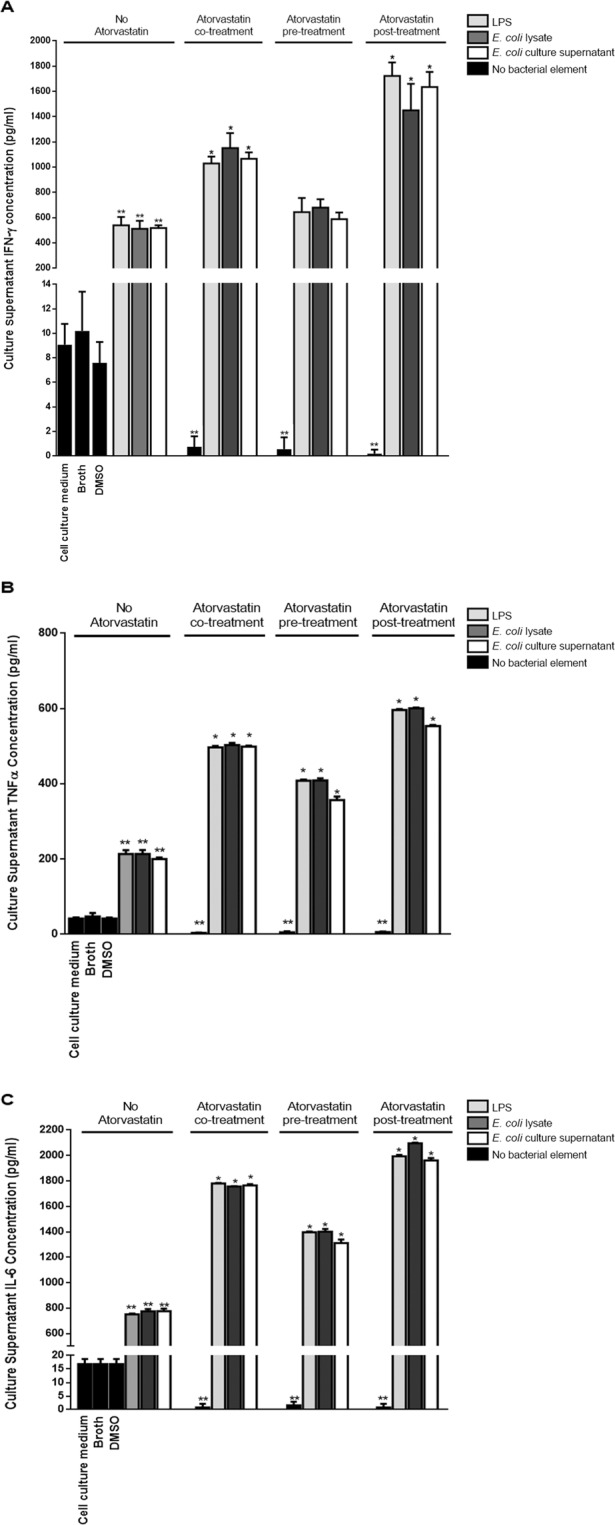
Proinflammatory cytokine levels in culture supernatants from mouse PBMCs incubated with bacterial components and treated with atorvastatin. Mouse PBMCs were cultured for 12 hrs at 37 °C prior to introduction of a bacterial treatment element [Lipopolysaccharide (LPS)/*E. coli* lysate/*E. coli* culture supernatant]. Cells were incubated with each bacterial element for another 12 hrs prior to culture supernatant collection for cytokine level analysis by ELISA. Atorvastatin was introduced 4 hrs before, along with or 4 hrs after introduction of the bacterial treatment element. Control treatments consisted of culture medium alone (LPS solvent), DMSO (atorvastatin solvent) or sterile Brain Heart Infusion (BHI) broth; these were added to cells that were cultured for 12 hrs at 37 °C. Atorvastatin control treatments were also included with the statin being added in a timeline similar to that used for the bacterial element-treated cells but in the absence of this latter type of treatment. (**A**) IFN-γ levels. (**B**) TNFα levels. (**C**) IL-6 levels. *indicates p < 0.05 compared to the respective *E. coli* treatment element in absence of atorvastatin; **indicates p < 0.05 compared to cells mock treated with vehicle (Culture medium for LPS, BHI broth for *E.coli* lysates or culture supernatant and DMSO for atorvastatin).

Consistent with our previous observations, a significant 7 to 10-fold decrease in the levels of IFN-γ was observed in culture supernatants of mouse PBMCs treated with atorvastatin compared to cells that were not exposed to this statin (Fig. 4). On the other hand, treatment with LPS, the *E. coli* cell lysate or *E. coli* culture supernatant led to an approximate 50-fold increase in the level of this cytokine compared to untreated cells. Compared to cells that were treated with LPS alone, a 2-fold increase (p = 0.0006) in IFN-γ levels was observed in cells receiving a co-treatment of LPS and atorvastatin while a 3-fold increase (p = 0.0001) was detected in cells that were treated with atorvastatin after being incubated with LPS. A rather similar trend and extent of increase was seen in culture supernatants from mouse PBMCs incubated with the *E. coli* cell lysate or *E. coli* culture supernatant and receiving a co- or post-treatment with atorvastatin. Mouse PBMCs that were pre-treated with atorvastatin prior to being incubated with a bacterial component did not show remarkable elevation of IFN-γ levels over PBMCs incubated with a bacterial component but not treated with atorvastatin. Worth noting is that the *E. coli* lysate and the *E. coli* culture supernatant both had a similar effect on IFN-γ levels to that caused by the LPS employed at an amount equivalent to that contained in the bacterial lysate or bacterial culture supernatant. This rather indicates that other toxins and components contained within or released from *E. coli* cells do not have an additive effect that contributes to the noted induction of IFN-γ. Similar observations were made upon testing for TNFα (Fig. 4B) and IL-6 (Fig. 4C) levels; while the various atorvastatin treatment regimens resulted in significantly decreased levels of these proinflammatory mediators, this inhibition was overcome by treatment with LPS, *E. coli* cell lysate or *E. coli* culture supernatant largely mirroring our *in vivo* observations. Hence, LPS is likely the *E. coli* component overcoming the atorvastatin-mediated inhibition of IFN-γ production.

**Figure 5:**
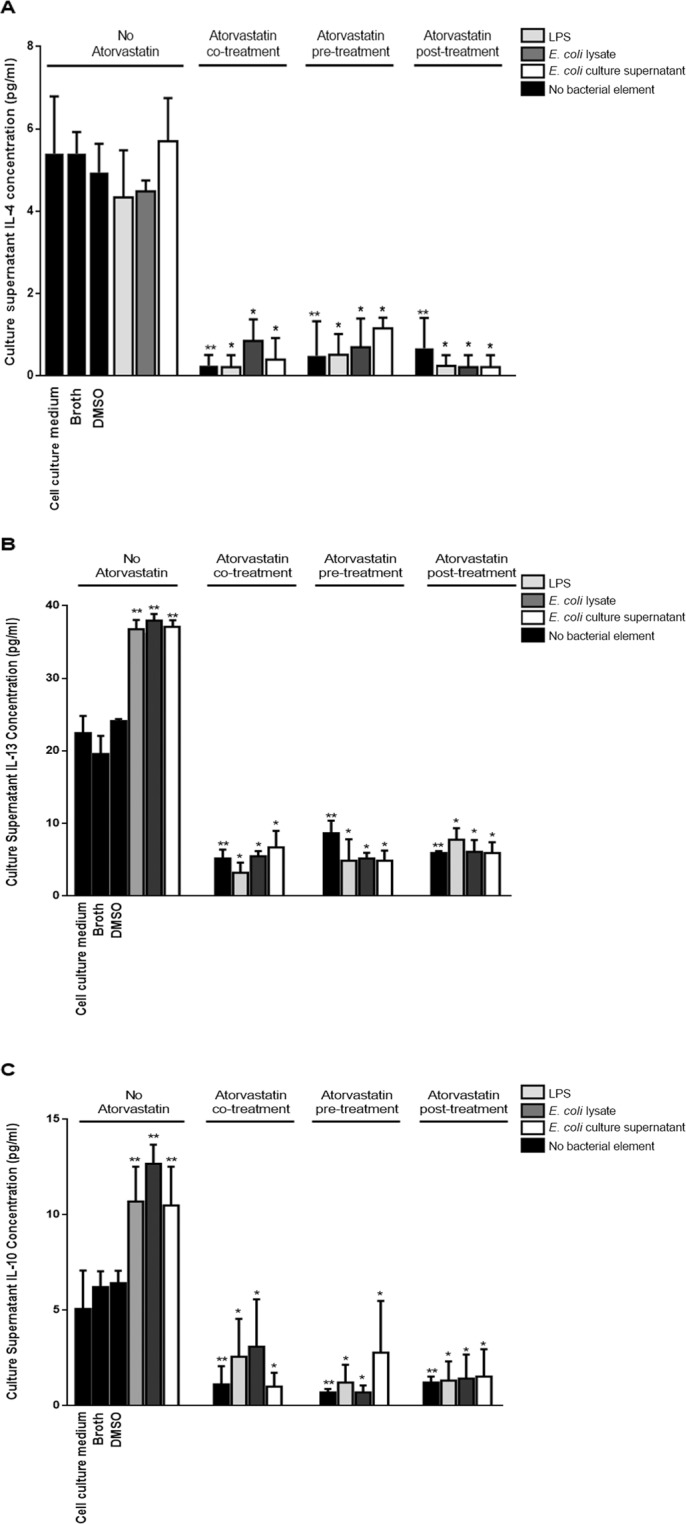
Prohumoral/regulatory cytokine levels in culture supernatants from mouse PBMCs incubated with bacterial components and treated with atorvastatin. Mouse PBMCs were cultured for 12 hrs at 37 °C prior to introduction of a bacterial treatment element [Lipopolysaccharide (LPS)/*E. coli* lysate/*E. coli* culture supernatant]. Cells were incubated with each bacterial element for another 12 hrs prior to culture supernatant collection for cytokine level analysis by ELISA. Atorvastatin was introduced 4 hrs before, along with or 4 hrs after introduction of the bacterial treatment element. Control treatments consisted of culture medium alone (LPS solvent), DMSO (atorvastatin solvent) or sterile Brain Heart Infusion broth; these were added to cells that were already cultured for 12 hrs at 37 °C. Atorvastatin control treatments were also included with the statin being added in a timeline similar to that used for the bacterial element-treated cells but in the absence of this latter type of treatment. (**A**) IL-4 levels. (**B**) IL-13 levels. (**C**) IL-10 levels. *indicates p < 0.05 compared to the respective *E. coli* treatment element in absence of atorvastatin; **indicates p < 0.05 compared to cells mock treated with DMSO.

Treatment with bacterial elements did not enhance IL-4 (Fig. 5A) production from mouse PBMCs over baseline but resulted in a moderate increase in those of IL-13 (Fig. 5B) and IL-10 (Fig. 5C). On the other hand, various treatment modalities with atorvastatin resulted in a significant decrease in the levels of these cytokines compared to mouse PBMCs that were not treated with atorvastatin again mirroring our *in vivo* observations; however, unlike our observations with IFN-γ, TNFα and IL-6 this inhibition by atorvastatin was not overcome by treatment with the bacterial elements.

## Discussion

Whether statins improve the outcomes of bacterial infection and sepsis has been a debatable issue with various studies indicating opposing data in addition to possible study biases. This is despite the fact that statins have been shown to exert direct antibacterial effects and that multiple animal model studies have reported improved outcomes for animals treated with statins and challenged with a bacterial agent or a bacterial component such as LPS^16–23^.

In the study at hand we report that atorvastatin administration markedly exacerbated the outcome of mouse infection with *E. coli* when this statin was given after the infection resulting in a decreased animal survival rate. Atorvastatin was given in 40 mg/Kg injections; this dose was employed since it is the minimum dose to induce immunomodulatory changes in BALB/c mice as we previously reported^15^. Using the United States Food and Drug Administration (FDA) mouse to human dose translation guidelines^24^ this dose is equivalent to that of 3.24 mg/Kg in humans. Although this is a higher dose than what is typically administered for treatment, comparable high doses have been previously employed in some subject groups^25^ and perhaps more relevantly, it would allow for examining the effects of multi-dosing which has been reported to cause spikes in cumulative blood levels of patients on atorvastatin^26^. The decreased animal survival rate we observed upon statin treatment of *E. coli*-infected mice was accompanied by a rather significant elevation in the level of serum IFN-γ, the proinflammatory cytokine. The decrease in survival, coinciding with an increase in IFN-γ levels is possibly more than just two concurrent outcomes resulting from atorvastatin treatment of *E. coli*-infected mice. The more elevated IFN- γ levels very likely contribute to the decline in the survival rate, due to the exacerbation of inflammatory reactions to a harmful degree.

We had previously reported that atorvastatin decreases the expression of IFN-γ in mice challenged with egg albumin and in *C. albicans*-infected mice^14^. Examining whether a bacterial component could overcome this inhibition showed that LPS was the likely culprit. LPS-harboring bacterial elements were capable of overcoming the inhibition exerted by atorvastatin on the production of proinflammatory mediators but humoral/regulatory mediators. Mice that were treated with the statin prior to infection had a survival rate that was similar to the group infected but not treated with atorvastatin in addition to a serum level of IFN-γ that was not as marked as the group infected and treated with a single dose or post-treated with atorvastatin. This likely indicates that serum levels of atorvastatin in the pre-treated group were not sufficient enough after ceasing drug treatments to trigger an LPS-driven lethal inflammatory response upon infection. The increase seen in IFN-γ in the groups treated with one dose of atorvastatin on the same day as infection or post-treated with this statin may be an indicator of enhanced cell-mediated immunity. Nevertheless, this would not be of benefit in an infection with an extracellular pathogen such as *E. coli*. Moreover, atorvastatin impairs the IFN-γ-induced expression of the major histocompatibility complex (MHC) class II molecules on multiple cell types which rather affects the extent of T-cell activation and any potential resultant response of the adaptive arm of immunity^27^. Atorvastatin has also been shown to decrease the expression of Toll-like Receptor (TLR4) in human monocytes^28^. With TLR4 being the pattern recognition receptor (PRR) that recognizes LPS, this could further compromise downstream responses to *E. coli* in mice. Levels of IL-4, an indicator of humoral immunity, which may have been beneficial in this case, were notably decreased upon treatment with atorvastatin in the presence or absence of infection.

Cerivastatin treatment has been reported to decrease TNF-α and IL-6 levels in mice administered LPS but not in mice challenged with *Salmonella enterica* serovar Typhimurium despite enhancing the survival rate of mice treated with either agent^20^. However, Matsumoto *et al*.^29^ have demonstrated that simvastatin enhances LPS-triggered proinflammatory responses from mouse macrophages including increased expression of IL-12p40 and TNF-α. Montero *et al*.^30^ have also shown that fluvastatin enhances the production of IFN-γ, among other proinflammatory cytokines, from *Mycobacterium tuberculosis*-treated human PBMCs while Aktas *et al*.^31^ have reported decreased IFN-γ in a mouse model of experimental encephalomyelitis treated with atorvastatin. Unlike our findings, Youssef *et al*.^32^ have shown that atorvastatin induces the production of IL-4 in immune cells from the mouse model of experimental encephalomyelitis. However, multiple reports have indicated decreased levels of IL-4 in lung tissues from statin-treated mouse models of asthma^33–35^.

A possible explanation may be that the administered statin interacts differently with the organism, on the cellular and molecular levels, depending on the presence or absence of an immune response. As previously reported, statins possess different degrees of antibacterial activity when assessed *in vitro*^36^, whereby the statin may be able to exert its function directly without being affected by other factors. On the other hand, as observed *ex vivo* in this study, atorvastatin significantly suppressed IFN-γ, TNFα and IL-6 levels but not when an *E. coli* component was present. The microenvironment was likely substantially different in the presence of a bacterial component with possible modified expression of various immunologically-relevant cell surface molecules, triggered or suppressed cellular functions and altered cytokine levels. With the diverse pleiotropic effects that atorvastatin has and the complexity of immune responses, the outcome of treating an infection with a statin appears to be unpredictable and quite variable depending on the challenge, the statin used and the experimental system among other conditions.

Therefore, any possible consideration of statins as agents used in the treatment, prevention or modulation of a bacterial infection or septic response should encompass the disparity in the possible outcomes and analyze whether any positive effects can outweigh the possible risks involved.

## Methods

### Assessing the effect of atorvastatin on the survival of *E. coli*-infected mice

To examine whether atorvastatin treatment affects the survival rate of *E. coli*-infected mice, 4 groups of male BALB/c mice 6 to 8 weeks of age were used with each group containing 9 mice. One group was infected with *E. coli* but not treated with atorvastatin, another group was infected and received a co-injection of atorvastatin, a third group was pre-treated with atorvastatin hence receiving one injection of this statin per day for 4 days prior to infection and a fourth group was treated with atorvastatin post-infection hence receiving one injection of this statin per day for 4 days after being infected with *E. coli*. Treatment regimen and times of administration implemented were extrapolated from our previous assessments of the effect of atorvastatin in mice^14,15^. Mouse study protocols were approved by the Institutional Animal Care and Use Committee (IACUC) at the American University of Beirut (AUB); all methods were performed in accordance with the relevant guidelines and regulations. Animals were obtained from the Animal Care Facility at AUB.

Mice were each infected with 8.48 × 10^5^ colony forming units (CFUs) of the previously described CDC 07–98 *E. coli* strain^37–39^ administered via the intraperitoneal route. The selected infectious dose is sub-LD50 in BALB/c mice which hence allowed for detecting variability in the frequency of death events induced by treatment with atorvastatin. The intraperitoneal route was chosen on one hand so as to establish a systemic infection and on the other hand since BALB/c mice are resistant to infection with the organism by oral gavage in the absence of additional treatments or manipulation that may confound the results of the study at hand. Prior to initiation of mouse experiments, the susceptibility of the utilized strain to atorvastatin was ensured using Clinical Laboratory Standards Institute (CLSI) guidelines.

Atorvastatin (Pfizer Inc., New York, NY) injections consisted of 40 mg/Kg given intraperitoneally. Atorvastatin and infectious agent injections each consisted of 0.25 ml. Mice were monitored for 14 days after infection for survival. Mouse spleens, hearts and livers were collected upon death and then homogenized and cultured to ensure systemic infection had occurred. Organs of all infected mice tested positive for the organism.

### Assessing the effect of atorvastatin on serum IFN-γ and IL-4 levels in *E. coli*-infected mice

To examine serum IFN-γ and IL-4 levels as markers of proinflammatory and prohumoral immune responses, respectively, in infected animals treated with atorvastatin, a different set of mice were treated as described in the survival assessment section above. On day 4 post-infection, 3 mice from each group were sacrificed by cardiac puncture. This day was selected for immune marker assessment since mouse death events escalated on day 5 post-infection; hence, this allowed examining cytokine levels one day prior. Mouse sera were pooled per group and the IFN-γ and IL-4 levels were determined using Single Analyte ELISArray Kits (Qiagen Inc., Valencia, CA) as per manufacturer recommendations. Triplicate assessment was performed for each marker.

### Examining the effect of atorvastatin on the production of proinflammatory and prohumoral/regulatory cytokines from mouse PBMCs treated with bacterial components

BALB/c mouse peripheral blood mononuclear cells (PBMCs) were separated and cultured in 1640 RPMI (Sigma-Aldrich Chemie GmbH, Munich, Germany) supplemented with 10% fetal bovine serum (Sigma-Aldrich) and 1% penicillin-streptomycin (Lonza, Basel, Switzerland) for 12 hrs at 37 °C prior to introduction of a bacterial treatment element [Lipopolysaccharide (LPS)/*E. coli* lysate/*E. coli* culture supernatant].

Cells were cultured in 96-well plates with each well containing 5 × 10^5^ cells in 250 μl of medium. LPS from *E. coli* O111:B4 (Invivogen, Toulouse, France) was added at a concentration of 100 ng/ml; hence 25 ng of LPS were added per well treated with this toxin. A total of 5.4 × 10^8^ *E. coli* cells is estimated to contain 28.6 μg of LPS after 4 hrs of culture in broth^40^; hence, for addition of an amount of *E. coli* lysate that corresponds to the 25 ng of LPS used alone, bacteria were cultured for 4 hrs in Brain Heart Infusion broth then were centrifuged, resuspended in culture medium, sonicated and then the amount of lysate equivalent to 4.72 × 10^5^ cells was added per treated well. On the other hand, *E. coli* cultured for 4 hrs is estimated to release 5.7 μg of LPS per ml of broth^40^; therefore, to add a volume of bacterial culture supernatant that corresponds to the 25 ng of LPS used alone, 4.39 μl of filtered *E. coli* culture supernatant were added per treated PBMC cell culture well. The sterility of the lysates and bacterial culture supernatant was verified by inoculation into Brain Heart Infusion broth and incubation at 37 °C prior to use in the cell culture experiments.

After being cultured at 37 °C for 12 hrs as indicated above, PBMCs to be treated with the bacterial elements were incubated with these elements for an additional 12 hrs prior to cell culture supernatant collection for cytokine testing. Atorvastatin at a concentration of 5 μM was introduced either 4 hrs before, along with or 4 hrs after introduction of the bacterial treatment element. Hence, the overall PBMC culture time was 24 hrs. This concentration of atorvastatin was used since it was previously demonstrated to inhibit T cell activation in stimulated human PBMCs^41^ as well as being inhibitory to IFN-γ and IL-4 release from mouse PBMCs in our studies.

Control treatments consisted of culture medium alone (LPS solvent), 0.05% dimethyl sulfoxide (DMSO) (atorvastatin solvent) or sterile Brain Heart Infusion broth (4.39 μl). These were added to cells that had been cultured for 12 hrs; control treatment supernatants were collected after another 12 hrs of incubation. Atorvastatin was also assessed in the absence of incubation with a bacterial element and introduced in a similar timeline to that of the co-, pre- and post-treatment samples. Experimental and control treatments were performed in triplicates.

Mouse IFN-γ, TNFα, IL-6, IL-4, IL-13 levels were determined in cell culture supernatants using Single Analyte ELISArray Kits (Qiagen). IL-10 levels were assessed using an Abcam kit (Cambridge, England).

### Statistical analysis

PASW Statistics 18 for Windows was used to perform the log-rank (Mantel-Cox) test and Kaplan-Meier survival analysis. The GraphPad t-test calculator was used to conduct unpaired t-tests for cytokine level comparisons. P < 0.05 was considered to indicate statistical significance.

## Data Availability

The datasets generated and analyzed in the current study are available from the corresponding author upon reasonable request.
